# Genetic characterization of a novel pheasant-origin orthoreovirus using Next-Generation Sequencing

**DOI:** 10.1371/journal.pone.0277411

**Published:** 2022-11-21

**Authors:** Yi Tang, Haiyang Yu, Xiaoning Jiang, Endong Bao, Dong Wang, Huaguang Lu

**Affiliations:** 1 College of Animal Science and Technology, Shandong Agricultural University, Taian, Shandong Province, China; 2 Tianjin Ringpu Bio-Technology Co, Ltd., Tianjin, China; 3 Wiley Lab / Avian Virology, Animal Diagnostic Laboratory, Department of Veterinary and Biomedical Sciences, The Pennsylvania State University, University Park, PA, United States of America; Bangladesh Agricultural University, BANGLADESH

## Abstract

A field isolate (Reo/SDWF /Pheasant/17608/20) of avian orthoreovirus (ARV), isolated from a flock of game-pheasants in Weifang, Shandong Province, was genetically characterized being a field variant or novel strain in our recent research studies in conducting whole genome sequencing by using Next-Generation Sequencing (NGS) technique on Illumina MiSeq platform. Among a total of 870,197 35-151-mer sequencing reads, 297,711 reads (34.21%) were identified as ARV sequences. The *de novo* assembly of the ARV reads resulted in generation of 10 ARV-related contigs with the average sequencing coverage from 1390× to 1977× according to 10 ARV genome segments. The complete genomes of this pheasant-origin ARV (Reo/SDWF /Pheasant/17608/20) were 23,495 bp in length and consist of 10 dsRNA segments ranged from 1192 bp (S4) to 3958 bp (L1) encoding 12 viral proteins. Sequence comparison between the SDWF17608 and classic ARV reference strains revealed that 58.1–100% nucleotide (nt) identities and 51.4–100% amino acid (aa) identities were in genome segment coding genes. The 10 RNA segments had conversed termini at 5’ (5’-GCUUUU) and 3’ (UCAUC-3’) side, which were identical to the most published ARV strains. Phylogenetic analysis revealed that this pheasant ARV field variant was closely related with chicken ARV strains in 7 genome segment genes, but it possessed significant sequence divergence in M1, M3 and S2 segments. These findings suggested that this pheasant-origin field variant was a divergent ARV strain and was likely originated from reassortments between different chicken ARV strains.

## Introduction

Viruses of the family *Reoviridae* have segmented dsRNA genomes and are classified into two subfamilies comprising a total of 15 genera [1]. The genus *Orthoreovirus* belongs to the subfamily *Spinareovirinae*, which currently has five species, including Mammalian orthoreovirus, Baboon orthoreovirus (BRV), Reptilian orthoreovirus (RRV), Nelson Bay orthoreovirus (NBV), and Avian orthoreovirus (ARV) [2, 3]. ARV is non-enveloped virus with icosahedral symmetry and contains a surface protein arranged in a double shell [4]. Virus particles have an average size of 70–80nm and are wrapped around 10 genomic fragments. Depending on their movement in electrophoresis, linear genomic fragments were molecularly divided into three different groups, including three large fragments (L1-L3), three medium fragments (M1-M3) and four small fragments (S1-S4) [5, 6]. Besides the tricistronic S1 segment, all ARV genome segments are monocistronic and all genome segments encode 8 structural proteins (λA, λB, λC, μA, μB, σA, σB and σC) and 4 nonstructural proteins (μNS, p10, p17 and σNS) [7]. Each ARV coding gene was flanked by 5’ and 3’ non-translated regions and the first seven bases (5’-GCUUUUU) and the last five bases (UCAUC-3’) of segment termini were found are highly conserved in known ARV strains [8].

ARV is a highly contagious virus involved in a variety of disease conditions or syndromes in poultry, of which viral arthritis/tenosynovitis is the most classic leg lameness or weakness seen in ARV-affected young broiler chickens [3, 9]. Since the 1980s, with the rapid development of the modern poultry industry, new symptoms or newly observed disease problems associated with ARV infections have been continuously reported, such as runting-stunting syndrome (RSS) [10], immunosuppression [11], hepatitis [12], malabsorption/maldigestion syndrome [13], respiratory disease [14], and enteric disease [15]. In recent years in Pennsylvania, commercial poultry flocks suffered viral arthritis/tenosynovitis have been increasingly diagnosed and field variant strains of the newly emerging ARVs were confirmed as the causative agent [16, 17]. During the same period, highly pathogenic ARV variants emerged in turkey in Midwestern United States [18, 19], as well as in other countries [18–20]. Similarly, an increasing number of cases of arthritis in broilers caused by ARV has occurred in China, and the ARV field variants remain higher genetic diversity and virulence in flocks, which caused considerably economic losses in the poultry industry. Most of the emerged variants showed common features of genome segments reassortments with historical ARV strains and high genetic diversity in σC genes [21].

Many research studies on ARV infections in various avian species, especially domestic poultry, have been well documented, such as broiler breeders [22], layer breeders [23], broilers [24], geese [25, 26], turkeys [27, 28], ducks [29–31], pigeons [32], and quails [33–35]. Although ARV transmissions commonly occur within and between avian species, the importance of wild birds as reservoirs of ARV transmission source to domestic poultry infections was not well studied until recent years due to the difficulty of wild bird sampling [36, 37]. Until now, there were only two pheasant cases of ARV infections were reported in 1990s, one case ARV strain was associated with hepatopathy symptoms and the other was associated with tenosynovitis [38], however, there is no previous report of pheasant being infected by ARV in China. In the present study, we report our findings of isolation and full-genome characterization of a novel pheasant-origin ARV field variant strain.

## Materials and methods

### Virus isolate

The pheasant ARV isolate (Reo/SDWF /Pheasant/17608/20) in this study was isolated from tendon tissue of a pheasant case with hepatopathy symptoms at 2–4 weeks of age. The ARV isolation was made in LMH cell cultures and produced giant or bloom-like cytopathic effect (CPE) cells, which were characteristic to ARV and confirmed positive for ARV by fluorescent antibody (FA) test using ARV conjugated antibody (ID No. 680 VDL 9501, NVSL, Ames, IA, USA) which described in our previous study [39]. The ARV cell culture material was subsequently tested negative for other avian viruses which could cause the pheasant leg lesions such as avian influenza virus, Newcastle disease virus, fowl adenovirus type 1 and rotavirus. This pheasant ARV was propagated in LMH cell cultures, tittered as 10^8.5^ TCID_50_/mL, aliquoted and stored at -80°C freezer for this study.

### RT-PCR and Sanger sequencing

Viral RNA extraction of the pheasant ARV was performed by using MiniBEST Universal RNA Extraction Kit (TaKaRa, Dalian, China) per the manufacturer’s instructions. Conventional RT-PCR reaction was carried out with P1/P4 primers which corresponding to 3’ end of S1 segment (σC gene) of ARV [39] by using the One Step RT-PCR Kit Ver.2 (TaKaRa, Dalian, China). RT-PCR products were observed by 1% agarose gel electrophoresis and the 1088bp band was purified by using gel extraction kit (OMEGA, D2500-02 Gel Extraction Kit, USA) following the manufacturer’s instructions. The concentration of the purified DNA was confirmed by using a NanoDrop™1000 (DeNovix DS-11, USA) spectrophotometer and then submitted to Personalbio for Sanger sequencing.

### Next-generation sequencing

NGS was carried out on a Miseq platform. Total RNA samples were processed by MiniBEST Universal RNA Extraction Kit (TaKaRa, Dalian, China) to build cDNA library. Briefly, the total RNA was fragmented into small pieces using magnesium divalent cations under elevated temperature [40]. The cleaved RNA fragments were copied into the first strand cDNA using reverse transcriptase (Invitrogen, Grand Island, NY, USA) and random primers. The second strand cDNA synthesis was followed using DNA polymerase I and RNase H but without the initial poly A enrichment step. Then these cDNA fragments were assessed by Direct Detect™ bioanalyzer system (DeNovix DS-11, USA) to test the fragments distribution. Thereafter, the prepared cDNA library was loading on the Miseq sequencer to get the raw NGS reads.

### *De novo* assembly of viral genome

The CLC Genomics Workbench V7.5.2 software (QIAGEN, Boston, MA, USA) was used for NGS raw data De novo assembling. Firstly, sequencing adaptors were trimmed off and contaminants sequences mapped to rRNA and mRNA reads were removed. The rest of clean reads were processed by software to get contigs. All ARV-related contigs were selected to build the full-length genome of ARV based on the BLASTN searching result. Each assembled segment was further upgraded by mapping back all NGS raw reads to the ARV-related contigs, and the consensus sequences were considered as the complete ARV genome.

### Sequence analyses

The modules of EditSeq and MegAlign of DNASTAR Lasergene 12 Core Suite (DNASTAR, Inc. Madison, WI, USA) were used for viral open reading frames (ORFs) prediction, amino acid (aa) translation, sequence alignment, and pair-wised sequence comparison. An online search program (http://blast.ncbi.nlm.nih.gov/Blast.cgi) identified the highest similarities between the studied ARV genome segment and the published sequences.

Sequencing coverage, mapped reads, and intra-host single-nucleotide variants (iSNVs) of each assembled contigs were calculated and visualized by CLC Genomic Workbench V7.5 software (QIAGEN, Boston, MA, USA). Phylogenetic analysis of genome segments were carried out by using the neighbor-joining method in MEGA CC program [41] and the bootstrap validation method with 1000 replications. The visualization of genome alignment was performed using the mVISTA (http://genome.lbl.gov/vista/mvista/submit.shtml) and the scale sequence was using studied pheasant-origin ARV genome. Genome sequences of 13 ARV reference strains were retrieved from Genbank for sequence comparisons (S1 Table).

## Results

### Sanger sequencing

One-step RT-PCR was used to test the pheasant ARV viral RNA using specific primers (P1/P4) based on the S1 gene. As a result, the 1088bp PCR product was successfully amplified, and sanger sequencing results of showed the pheasant ARV strain (MZ561700) has about 88% nucleotides homology to those of other novel GoAstV strains, ARV strain in GenBank (L07069). In the next-generation sequencing analysis, the contigs of ARV were clearly mapped, whereas draft contigs to other-related viruses were not mapped.

### Analyzing the NGS raw data

**Fig 1:**
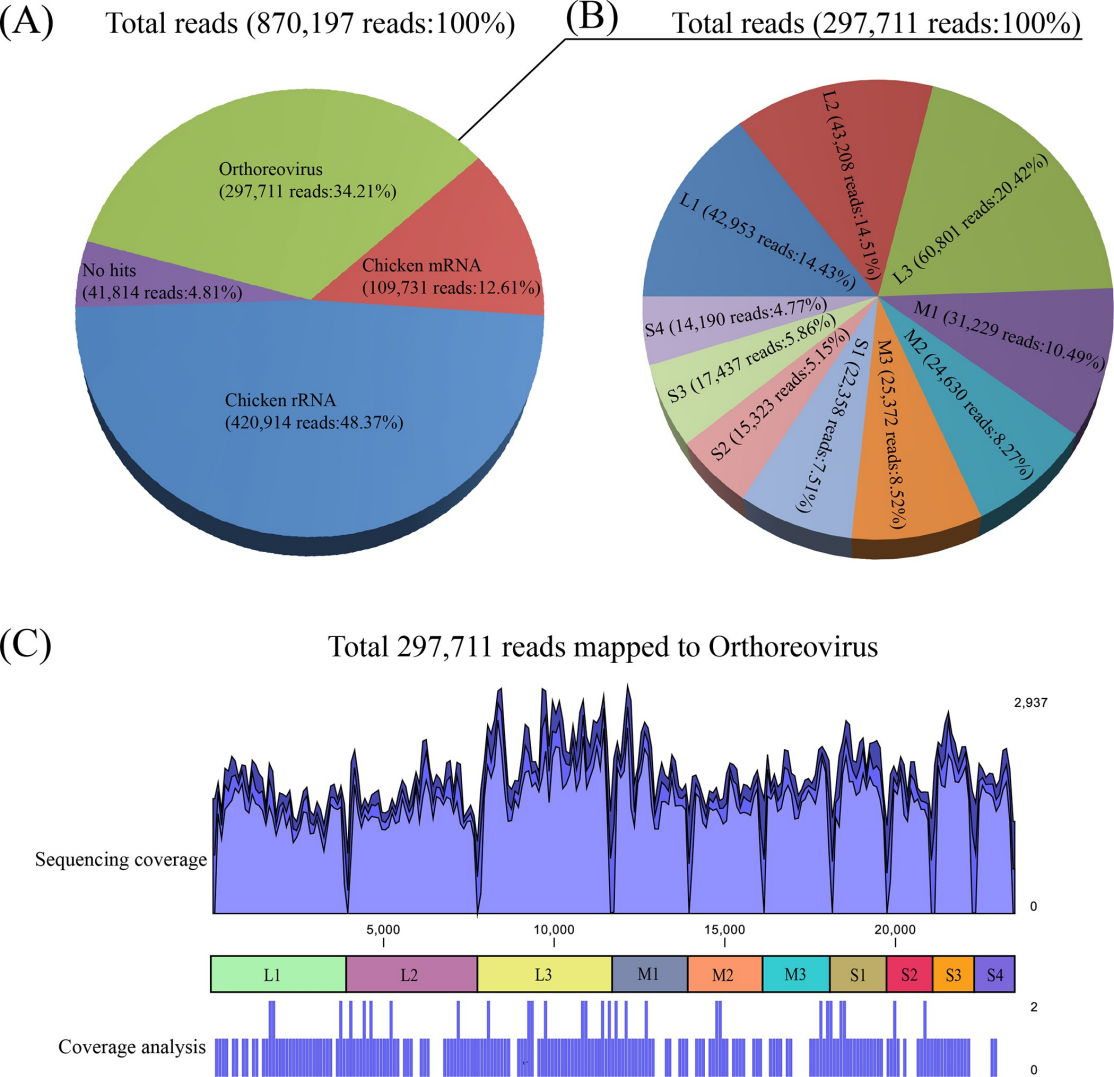
The illustrations of the homology search results for NGS reads and the sequencing coverage analysis. A: Total NGS reads homology search result; B: The remapping of NGS raw reads to ARV 10 segments; C: The result of sequencing coverage analysis.

A total of 842,235 sequencing reads of 35-151-mer were generated on Miseq sequencer from extracted viral total RNA. The final NGS sequence data from the viral stocks output file in fastq format was 79.3Mb in size. Low-quality reads, trim poly-T tails and adapter sequences were processed by quality control (QC) filters of the Miseq platform for removal, A further screening of the NGS reads was carried out to remove the non-research target readings that were similar to mRNA or rRNA sequences. As a result, 420,914 reads (48.37%) were identified to be the chicken rRNA source and 109,731 reads (12.61%) to be the chicken mRNA source (Fig 1A). The residual 339,525 clean reads were further analyzed using a BLASTN procedure, which further divided into no hits group (41,814 reads, 4.81%) and orthoreovirus group (297,711 reads, 34.21%) (Fig 1A).

### De novo assembly of viral genomes

After the assembly of no hits reads and orthoreovirus by *de novo* assembler of CLC Genomics Workbench software, a total of 72 contigs were generated with length from 150nt to 3958nt. The total reads counts of assembled contigs were found from 2 to 102,404 and the average coverage were from 1.08× to 6,506.34×. By searching online using BLASTN, 10 of the 72 contigs (Table 1) were identified as ARV-related contigs, ranging in length from 1192nt to 3958nt. The highest similarity search of 10 ARV contigs in Genbank revealed that all contigs had different homology (88.0%-96.6%) to other published reference ARV strains (Table 1). The initial alignment of assembled contigs and most homology reference ARV segments indicated the full-length of 10 ARV segments including the 5’ and 3’ termini were individually targeted by 10 ARV contigs.

**Table 1:** General genome features of a pheasant-origin avian orthoreovirus (ARV), the novel field variant of pheasant ARV (Reo/SDWF/Pheasant/17608/20).

Contig Length (bp)	Contig name	Highest similarity ARV strain in GenBank	Identities (%)	SNVs	Mapped reads	Average coverage	Encoded protein	Length of the(bp)		
								5’ end	ORF	3’ end
3958	L1	919 strain; segment L1, lambda A gene (AY641739)	96.6	1	42953	1390	λA(core shell)	20	3882	56
3829	L2	AVS-B strain; segment L2, lambda B gene (FR694192)	96.3	0	43208	1439	λB(core RdRp)	13	3780	36
3907	L3	138 strain; segment L3, lambda C gene (EU707937)	93.6	0	60801	1977	λC(core turret)	12	3858	37
2283	M1	138 strain segment, M1, muA gene (AY557188)	88.2	0	31229	1730	μA(core NTPase)	13	2199	72
2158	M2	Reo/PA/Broiler/05682/12 strain, segment M2, muB gene (KM877329)	99.4	6	24630	1445	μB(outer shell)	29	2031	98
1996	M3	1017–1 strain, segment M3, muNS gene (AY573905)	99.5	0	25372	1564	μNS(NS factory)	24	1908	64
1644	S1	Somerville 4 strain, segment S1, p10, p17 and sigma C genes (L07069)	88.0	0	22358	1721	p10(NS FAST)	22	300	33
							p17(NS other)		441	
							σC(outer fiber)		981	
1324	S2	526 strain, segment S2, sigma A gene (KF741703)	89.1	0	15323	1455	σA(core clamp)	15	1251	58
1202	S3	Reo/PA/Broiler/05682/12 strain, segment S3, sigma B gene (KM877333)	91.5	0	17437	1710	σB(outer clamp)	30	1104	68
1192	S4	526 strain, segment S4, sigma NS gene (KF741705)	94.3	0	14190	1476	σNS(NS RNAb)	23	1104	65

### Sequencing coverage

By mapping back all NGS raw reads to the 10 assembled ARV contigs, the mapped reads and sequencing coverage of different genome segments were finally obtained. Although the mapped reads of each segment were various from 14,190 to 60,810, they were still positively correlated with the contig length (Table 1, Fig 1B). Sequencing coverage for each genome fragment averaged from 1390× to 1977×, indicating that there is sufficient redundancy to identify the PA136491 pheasant ARV genome. The sequencing coverage of each ARV fragment is illustrated by the wave-chart (Table 1, Fig 1C), with a peak (2937×) appearing in the L3 fragment.

Sequencing coverage and intra-host single nucleotide variants (iSNVs) were further analyzed using the resequencing analysis module of the CLC Genomic Workbench software. As a result, regions of high sequencing coverage were found throughout the ARV genome (Fig 1C) by setting above 10 bp as the date aggregation value and a total of 7 iSNVs were determined in L1 and M2 segments (Table 1) by setting the sequencing error correction value of 0.4%.

### General genome information

Finally, the complete sequences of 10 fragments of the pheasant antiretroviral genome were obtained and deposited into Genbank (MZ561694 to MZ561703). The SDWF17608 ARV genome fragment ranges from 1192bp (S4) to 3958bp (L1), with a total size of 23,495bp. The GC content displays variation between different genome segments, from 47% to 52%. There were 12 viral proteins encoded by one tricistronic segment (S1) and nine monocistronic segments. ORFs range in length from 3882 bp (λA) to 300 bp (p10), which were similar to the general characteristics of published ARV strains. Although the size of most proteins was identical between this pheasant ARV and the reference ARV strains, but the pheasant ARV non-structural protein p10 encoding gene on S1 segment showed heterogeneity.

**Fig 2:**
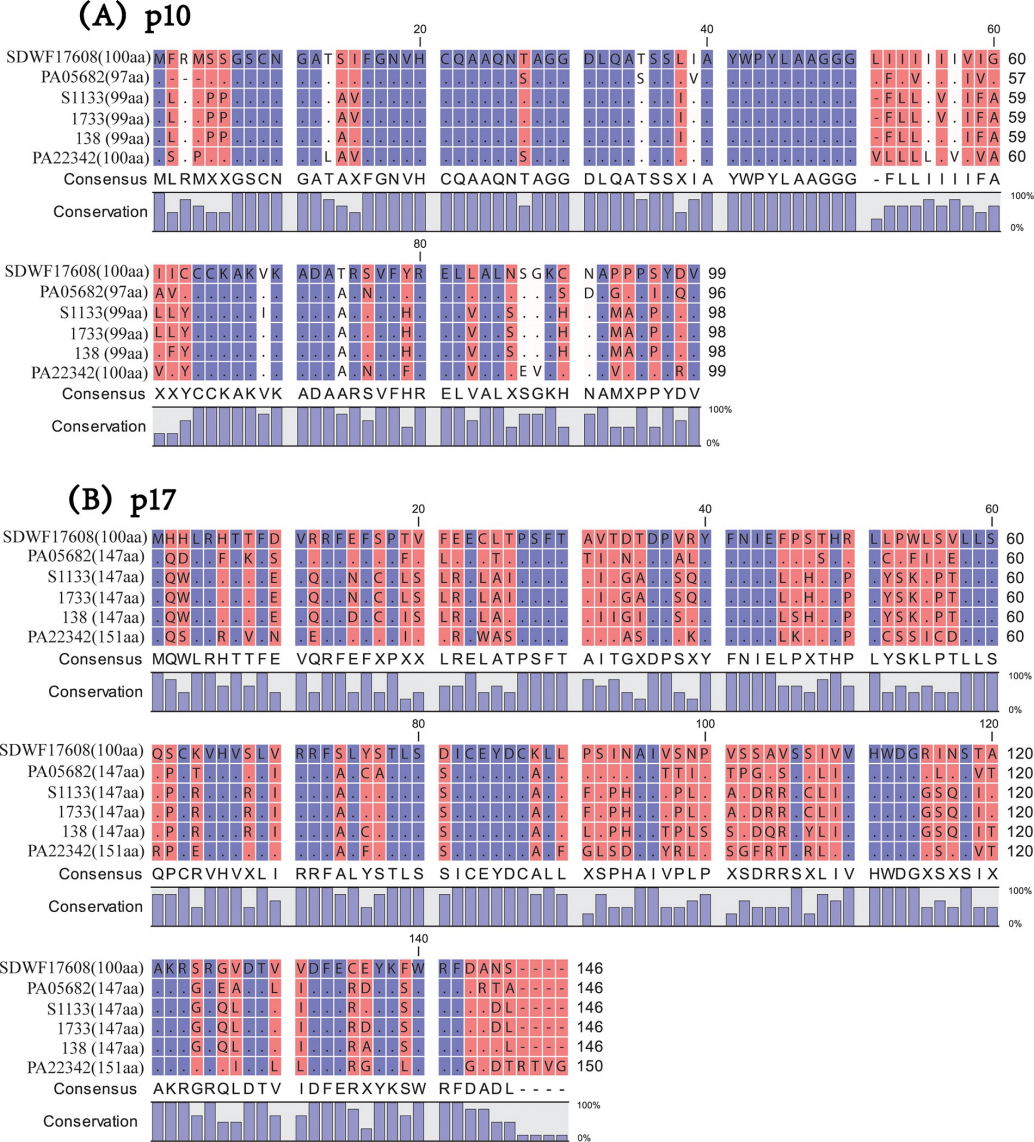
Amino acid alignment of p10 and p17 protein. A: The p10 protein of Reo/SDWF /Pheasant/17608/20 align with the homologous protein of Reo/PA/Broiler/05682/12 (or PA05682), S1133, 1733, 138 and Reo/PA/Turkey/22342/13 (or PA22342) strains; B: The p17 protein of Reo/SDWF /Pheasant/17608/20 align with the homologous protein of Reo/PA/Broiler/05682/12 (or PA05682), S1133, 1733, 138 and Reo/PA/Turkey/22342/13 (or PA22342) strains.

The p10 gene of the pheasant ARV was 300bp (100 aa) in length, which was larger than that of chicken ARV strains of PA05682 (291 bp, 97 aa), S1133 (297 bp, 99 aa), 1733 (297 bp, 99 aa) and 138 (297 bp, 99 aa) (Fig 2); whereas the p10 gene was the same between the pheasant ARV and the PA22342 turkey ARV. The untranslated regions (UTRs) were sited at the 5’ and 3’ termini of each genome segment with length of 12-30bp (5’ UTRs) and 33-98bp (3’ UTRs). The highly conserved terminal sequences of 5’ UTR (5’-GCUUUU-3’) and 3’ UTR (5’-UCAUC-3’) of the pheasant ARV were undistinguishable from other ARV reference strains, but distinguishable from the non-ARV reference strains (Table 2).

**Table 2:** Sequence identities between the pheasant ARV (Reo/SDWF/Pheasant/17608/20) and reference strains of *Orthoreovirus* genus members.

ARV Reference Strain	The pheasant ARV (Reo/SDWF/Pheasant/17608/20)																			
	λA		λB		λC		μA		μB		μNS		σA		σB		σC		σNS	
	nt	aa	nt	aa	nt	aa	nt	aa	nt	aa	nt	aa	nt	aa	nt	aa	nt	aa	nt	aa
PA05682	86.5	97.5	87.8	97.5	88.1	95.7	86.7	96.3	99.6	99.7	80.5	91.4	88.2	98.6	90.9	97.6	64.1	60.2	91.2	98.4
S1133	91.2	98.1	83.3	96.4	72.7	84.3	88.2	97.3	84.6	94.7	80.8	91.8	90.2	96.9	85.1	94.6	54.2	49.5	81.6	93.8
1733	91.4	98.5	83.5	96.7	72.9	84.2	88.3	97.4	84.6	94.7	81.0	92.1	90.3	97.8	85.3	95.1	54.3	49.2	81.8	94.0
138	88.9	98.3	89.3	98.1	91.7	96.4	89.9	97.0	89.5	97.3	80.3	91.8	89.9	98.1	90.0	97.6	55.7	50.8	89.1	96.7
PA22342	84.4	96.8	83.3	95.1	83.7	90.6	86.7	96.3	84.1	93.5	80.4	90.1	88.0	97.1	71.7	77.2	55.6	51.4	78.9	91.3
J18	77.2	94.7	76.1	91.0	70.4	79.0	74.4	87.2	75.8	89.8	71.3	81.0	76.9	92.1	64.7	68.2	42.5	29.7	78.4	90.5
BRoV	53.8	51.0	44.5	45.2	40.1	26.0	45.8	34.3	50.8	47.0	38.6	22.0	45.7	33.3	41.7	21.7	NA	NA	44.6	35.5
BRV	55.0	51.5	53.6	50.2	39.7	26.5	45.8	33.6	49.6	38.8	35.8	23.4	43.0	28.9	36.0	16.9	NA	NA	53.6	48.9
NBV	66.1	72.7	64.6	70.5	47.2	39.3	52.6	46.7	64.0	68.0	47.8	38.8	59.6	60.8	47.9	37.6	36.7	40.2	54.1	49.5
PuV	67.0	73.5	64.2	71.0	47.8	39.3	52.7	46.8	63.5	68.7	47.9	39.0	58.5	60.2	46.5	36.5	35.8	24.5	42.5	27.1
MRV1	49.8	43.1	55.1	54.4	41.5	27.8	44.2	28.3	51.8	45.8	41.0	23.5	43.8	29.1	40.1	19.4	34.6	19.0	44.0	23.1
MRV2	50.2	42.9	55.7	54.5	40.6	27.3	45.1	28.1	52.7	45.6	42.8	23.6	44.2	29.1	38.7	19.4	35.7	21.7	43.9	22.9
MRV3	49.9	42.7	55.4	54.4	41.2	27.3	44.6	27.9	52.3	45.6	41.7	23.5	43.5	29.1	39.6	19.2	30.6	19.0	42.2	22.6

Note: aa = amino acid sequence; nt = nucleotide sequence; NA = sequence not available in Genbank.

### Sequence comparisons

In homologs comparison of the nucleotide (nt) and aa sequences, we found that the pheasant ARV had various similarities ranging from 42.5% nt to 99.6% nt and 29.7% aa to 99.7% aa (Table 3), which were obtained in pairwise comparisons with six ARV reference strains and seven non-ARV orthoreovirus strains (S1 Table). In addition, the comparison results of σC encoding genes revealed that was the most divergent gene between the SDWF17608 and other reference ARV strains by showing the extremely low nt and aa identities (nt: 42.5–64.1%; aa: 29.7–60.2%). In comparisons with different avian species-origin ARVs, the pheasant ARV showed highest identity with PA05682 broiler ARV in μB-, σA-, σB- and σNS-encoding genes (nt: 90.9–99.6%; aa: 97.6–99.7%), high identity with ARV 1733 strain in λA- and μNS-encoding genes (nt: 81.0–91.4%; aa: 92.1–98.5%), and high identity with ARV 138 strain in λB-, λC- and μA-encoding genes (nt: 89.3–91.7%; aa: 96.4–98.1%). The SDWF17608 showed moderate identities to PA136491 turkey-derived ARVs (nt: 71.7–88.0%; aa: 77.2–96.8%) and low identity (nt: 64.7–78.4%; aa: 68.2–94.7%) with the J18 duck-origin ARV, and moderate to high identities with other ARV reference strains in the nonstructural protein p10 (nt: 70.1–88.2%; aa: 74.7–96.0%) and p17 (nt: 62.4–86.4%; aa: 61.2–99.3%) which encoded by S1 segment.

**Table 3:** Comparison of segment 5’ and 3’ non-coding regions of the pheasant ARV (Reo/SDWF/Pheasant/17608/20) with reference strains of *Orthoreovirus* genus members.

Orthoreovirus species	Host	Terminal region sequences	
		5’ end	3’ end
Reo/SDWF/Pheasant/17608/20	Pheasant	GCUUUU^U^/_C_	UA^U^/_C_UCAUC
Reo/PA/Broiler/05682/12	Broiler Chicken	GCUUUU^U^/_C_	UA^U^/_C_UCAUC
S1133	Broiler Chicken	GCUUUUU	UA^U^/_C_UCAUC
1733	Broiler Chicken	GCUUUU^U^/_C_	UA^U^/_C_UCAUC
138	Broiler Chicken	GCUUUUU	UAUUCAUC
Reo/PA/Turkey/22342/13	Turkey	GCUUUUU	UAUUCAUC
J18	Muscovy Duck	GCUUUUU	UA^U^/_C_UCAUC
Broome virus (BRoV)	Little Red Flying Fox	GUCAA	UCAUC
Baboon orthoreovirus (BRV)	Yellow Baboon	GUAAA	UCAUC
Nelson bay virus (NBV)	Grey-headed Flying Fox	GCUUUA	UCAUC
Pulau virus (PuV)	Fruit Bat	GCUUUA	UCAUC
Mammalian orthoreovirus (MRV)-1	Mink	GCUA	UCAUC
Mammalian orthoreovirus (MRV)-2	Human	GCUA	UCAUC
Mammalian orthoreovirus (MRV)-3	Masked Civet Cats	GCUA	UCAUC

The alignment of p10 (Fig 2A) and p17 (Fig 2B) proteins revealed that only p10 protein had a highly conserved region (aa 16 to 50) among the SDWF17608 pheasant ARV and other reference ARV strains.

In comparison with seven non-ARV orthoreovirus strains, the pheasant SDWF17608 shared much lower sequence identities (nt: 30.6–67.0%; aa: 19.0–73.5%) than those within ARV species, and their high homology was confirmed to be NBV in λB-, μA-, μB-, σA-, σB-, σC- and σNS-encoding genes (nt: 36.7–64.0%; aa: 37.6–68.0%) and PuV in λA-, λC- and μNS-encoding genes (nt: 47.8–67.0%; aa: 39.0–73.5%).

For UTRs comparison, all ARV strains shared a common motif of first six bases in 5’ UTR (Table 3). The seventh base of 5’ UTR was conserved in S1133, 138, PA22342 and J18 strains, but showed heterogeneity in other ARV strains, including SDWF17608 strain. The 5’ UTR of non-ARV strains was distinct from ARV strains and only showed some conserved regions within species (Table 3). In 3’ UTR, the UAUUCAUC-3’ motif was shared by all ARVs, although the second uracil may be replaced by cytosine base in some segments (Table 3). The last five bases of 3’ UTR (UCAUC-3’) were shared by all orthoreovirus members (ARVs and non-ARV orthoreoviruses) and considered as the genus common motif.

### Phylogenetic analysis

**Fig 3:**
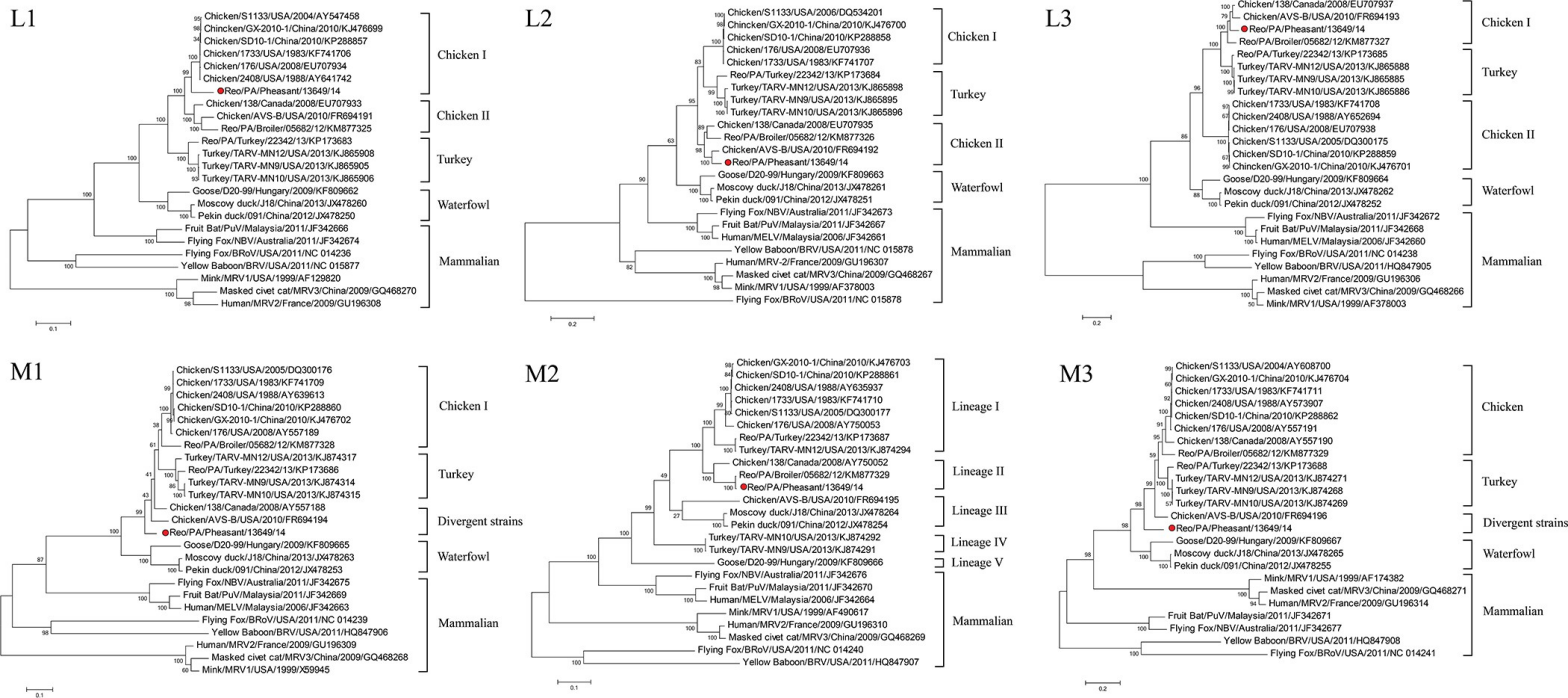
Phylogenetic trees constructed by avian orthoreovirus (ARV) based on nucleotide sequences of the L-class, M-class and σ-class homologous genome segments or genes. Note: The Reo/SDWF /Pheasant/17608/20 strain was marked with a red color dot.

By the rooted maximum likelihood phylogenetic analysis, the phylogenetic trees of the evolutionary relationships between the pheasant ARV of SDWF17608 and other orthoreovirus members were confirmed. Fig 3 is the phylogenetic trees generated by aligning their nt sequences of the three L (L1-L3) and three M (M1-M3) genome segments and four σ-class genes. For L-class segments analysis, five host-associated groups were formed by the SDWF17608 strain and other reference strains in all three L segments (L1-L3).

All ARV strains were divided into three subclades, chicken ARV, a turkey ARV, and a waterfowl ARV, while all non-ARV strains from mammalian species were more distantly related to ARV strains in a separate branch The SDWF17608 strain was closely related to some historical ARV strains of chicken group I in L1 segment (Fig 3, L1), but clustered with pathogenic ARV strains of chicken group II in L2 and L3 segments (Fig 3, L2 and L3). whereas DAstV-1 formed another clade with pathogenic ARV strains in in L2 and L3 segments.

For M-class segments analysis, host-associated groups were also observed in M1 and M3 trees, but the SDWF17608 strain evolved distant from the grouped ARV strains and fell into the divergent strains group (Fig 3, M1 and M3). The M2 segment of the SDWF17608 strain classified as a lineage 2 member, together with the PA05682 ARV variant and the classic ARV 138 (Fig 3, M2).

The phylogenetic tree analysis based on σ-class gene indicated the variety relationships between SDWF17608 strain and the reference strain. Overall, the ARV and non-ARV orthoreovirus strains were clearly divided into two main branches, and antiretroviral strains can be further divided into distinct subgroups or clusters. (Fig 3).

For σB and σNS genes, SDWF17608 strain evolved distantly from all vaccine strains or classic strains and grouped with the newly emerged pathogenic ARV variants (Fig 3, σB and σNS). In contrast, the σA gene of SDWF17608 strain was not grouped with any classic or pathogenic ARVs, but evolved as a divergent variant strain (Fig 3, σA). As shown in Fig 3, the σC was the most diverse gene among all 10 ARV segment genes.

Phylogenetic analysis of σC genes among all ARV strains resulted in generation of five genotyping clusters, in which the SDWF17608 strain was grouped into genotyping cluster 5 similar to German and Israeli ARV strains [42].

### Whole genome alignment

**Fig 4:**
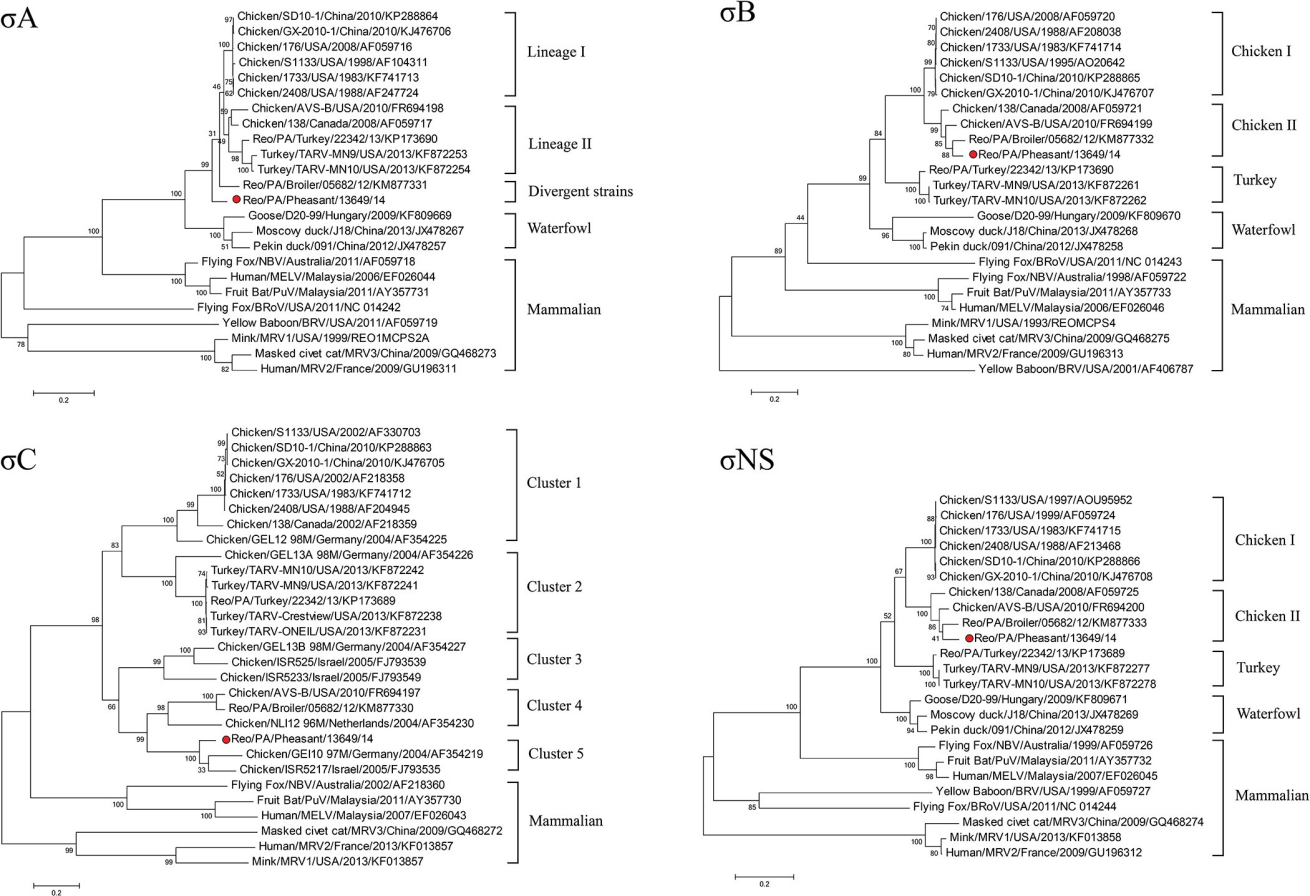
The mVISTA method for whole genome nucleotide alignment. Alignment result of the Reo/SDWF /Pheasant/17608/20 in comparisons with the Reo/PA/Broiler/05682/12 (or PA05682), S1133, 1733, 138, Reo/PA/Turkey/22342/13 (or PA22342) and J18 strains was illustrated; Areas in pink color represent ≥ 90% similarities; and areas in white represent < 90% similarities. The scale bar measures approximate length of the concatenated genome.

Whole-genome analysis of pheasant ARV and reference ARV strains was visualized by using the mVISTA online program (Fig 4). Eight of the 10 genome segments of the pheasant ARV and ARV 138 strains were broadly genetically related, with exclusion for the M3 segment and most 3’ regions of the S1 segment which corresponding to σC gene. Nevertheless, the M3 and S1 fragments of SDWF17608 strain showed the highest identity to the ARV S1133 and PA05682 strains, respectively. Except for the high similarity in the M2 segment between PA22342 turkey ARV and SDWF17608, most of the other regions are of moderate sequence identity. The duck-origin J18 ARV shared low sequence identities with SDWF17608 strain throughout their genomes, and an even lower identity was observed in S1 segment (<50%), which indicated no segment reassortment between the duck-origin ARV and the pheasants-origin ARV.

## Discussion

ARV as a causative agent of the highly pathogenic and contagious viral arthritis disease of chicken, and ARV-related diseases continue to emerge and expand to other domestic and avian species. The newly emerging ARV field variants mainly affect broiler, layer and turkey productions in the past decade [16, 17, 43, 44].

Numerous studies of the newly emerged ARV field variants suggest that a variety of ARV pathotypes or genotypes were circulating among different flocks and poultry spices [23, 45–47].

Although pheasant production is a relatively small-scale poultry business such as the game birds, pheasants can serve as a potential host repository for ARV transmissions and/or ARV genome reassortments to other avian species. This study provides data for the full-genome characterization of pheasant ARV, a novel pheasant ARV variant. By using the method of pairwise sequence comparison, we confirmed that all 10 genomic segments of SDWF17608 strain were nearly identical to the homologous segments of the reference ARVs.

The conserved regions at the 5’ and 3’ ends of the positive strand are the same as the ARV members, but there are some differences at the 5’ end with the non-ARV reference strains, indicating that the genome segments of SDWF17608 strain were all classified into ARV species and did not undergo fragment reassortment with non-ARV strains [48].

The comparison of all coding genes showed that SDWF17608 strain was most closely related to the chicken-origin ARV strains (PA05682 and 138), and the sequence similarity of μB gene with PA05682 broiler antiretroviral strain was the highest (nt: 99.6%; aa: 99.7%). The above results indicated that the PA05682 strain was involved in the ARV genome rearrangement to generate the SDWF17608 strain. As the major outer capsid protein, μB is responsible for virus entry/un-coating and transcriptase activation [49]. The specific sequence of μB gene may be required for an efficient establishment of a successful ARV infection in a specific avian host. The comparison results showed that the SDWF17608 strain was highly identical to the PA05682 broiler antiretroviral μB gene, suggesting that although the SDWF17608 strain was derived from pheasants, it may have the ability to infect other birds. In contrast, σC was the most divergent gene with low sequence identity to the compared strains (nt: <64.1%; aa: <60.2%), which was mainly because the σC gene was the most variable protein in the ARV [50] and showed the less interaction with other viral proteins [42]. In addition, the σB gene encodes the viral in vitro capsid protein and is generally of low homology among different ARV strains. Interestingly, the comparison of σB gene of the SDWF17608 strain in the present study showed host-related identity values, suggesting that the σB gene could be a candidate for a genetic marker to distinguish ARVs from various avian hosts.

The SDWF17608 strain expressed a high degree of diversity with classical ARV strains in its M1, M3 and S2 segments. The SDWF17608 strain was most closely related to PA05682 broiler ARV, and 138 strains were further confirmed by phylogenetic analysis in sequence comparison.

NGS is an important tool for assessing genetic diversity, providing high sequencing coverage for population samples such as viruses [51]. In the present study, the sequencing coverage of SDWF17608 strain was observed from 1390× to 1977× on average for different genomic segments. Such high sequencing coverage allowed us to calculate the reliable numbers of iSNVs in viral genome [52, 53].

In this study, the iSNV information provided us a number of heterogeneous nucleotide positions of the SDWF17608 strain. Six of the 7 iSNV positions were found in the M2 segments of the μB gene, likely due to the location of the μB protein in the outer capsid leading to more immune selection for the μB gene. However, no heterogeneous sites were observed in the σC and σB genes, suggesting a lower level of spontaneous mutation in these two outer capsid proteins in the pheasant ARV strain (SDWF17608).

In conclusion, we have completed the whole-genome sequencing characterizations for this novel pheasant ARV (Reo/SDWF/pheasant/17608/20) through NGS technology on the Illumina Miseq platform. These findings provide scientific genomic data to better understand the genomic evolutionary relationships between different avian species-origin ARVs.

## Supporting information

S1 Table Genbank accession numbers of full genome segments of avian orthoreovirus (ARV) reference strains: PA05682, S1133, 138, 1733, 176, J18, BRoV, BRV, NBV, PuV, MRV1, MRV2 and MRV3. (DOCX)

## References

[1] Adams MJ, Lefkowitz EJ, King AMQ, Bamford DH, Breitbart M, Davison AJ, et al. Ratification vote on taxonomic proposals to the International Committee on Taxonomy of Viruses (2015). Arch Virol. 2015;160: 1837–1850. doi: 10.1007/s00705-015-2425-z 25913692

[2] Spandidos DA, Graham AF. Physical and chemical characterization of an avian reovirus. J Virol. 1976;19: 968–976. doi: 10.1128/JVI.19.3.968-976.1976 987252 PMC354937

[3] van der Heide L. The History of Avian Reovirus. Avian Dis. 2000;44: 638. doi: 10.2307/1593104 11007012

[4] Joklik WK. Structure and function of the reovirus genome. Microbiological Reviews. 1981. doi: 10.1128/mr.45.4.483-501.1981 7035855 PMC281525

[5] Nick H, Cursiefen D, Becht H. Structural and growth characteristics of two avian reoviruses. Arch Virol. 1975. doi: 10.1007/BF01317969 170888

[6] Varela R, Benavente J. Protein coding assignment of avian reovirus strain S1133. J Virol. 1994. doi: 10.1128/JVI.68.10.6775-6777.1994 8084013 PMC237102

[7] Varela R, Martínez-Costas J, Mallo M, Benavente J. Intracellular posttranslational modifications of S1133 avian reovirus proteins. J Virol. 1996. doi: 10.1128/JVI.70.5.2974-2981.1996 8627773 PMC190156

[8] Su YP, Su BS, Shien JH, Liu HJ, Lee LH. The sequence and phylogenetic analysis of avian reovirus genome segments M1, M2, and M3 encoding the minor core protein μA, the major outer capsid protein μB, and the nonstructural protein μNS. J Virol Methods. 2006;133: 146–157. doi: 10.1016/j.jviromet.2005.10.031 16337282

[9] Sterner FJ, Rosenberger JK, Margolin A, Ruff MD. In vitro and in vivo characterization of avian reoviruses. II. Clinical evaluation of chickens infected with two avian reovirus pathotypes. Avian Dis. 1989. doi: 10.2307/1591119 2549942

[10] Goodwin MA, Davis JF, McNulty MS, Brown J, Player EC. Enteritis (so-called runting stunting syndrome) in Georgia broiler chicks. Avian Dis. 1993. doi: 10.2307/1591672 8363509

[11] Jones RC, Savage CE. Effects of experimental immunosuppression on reovirus-induced tenosynovitis in light-hybrid chickens. Avian Pathology. 1987. doi: 10.1080/03079458708436354 18766593

[12] Mandelli G, Rampin T, Finazzi M. Experimental reovirus hepatitis in newborn chicks. Vet Pathol. 1978. doi: 10.1177/030098587801500411 211695

[13] Page RK, Fletcher OJ, Rowland GN, Gaudry D, Villegas P. Malabsorption Syndrome in Broiler Chickens. Avian Dis. 1982;26: 618. doi: 10.2307/1589910 7150147

[14] Petek M, Felluga B, Borghi G, Baroni A. The Crawley agent: An avian reovirus. Archiv für die gesamte Virusforschung. 1967;21: 413–424. doi: 10.1007/BF01241740 5600964

[15] Dutta SK, Pomeroy BS. Isolation and characterization of an enterovirus from baby chicks having an enteric infection II. Physical and chemical characteristics and ultrastructure. Avian Dis. 1967. doi: 10.2307/1588321 4291487

[16] Tang Y, Lu H. Genomic characterization of a novel avian arthritis orthoreovirus variant by next-generation sequencing. Arch Virol. 2015. doi: 10.1007/s00705-015-2547-3 26234183

[17] Wang L, Fu S, Cao L, Lei W, Cao Y, Song J, et al. Isolation and identification of a natural reassortant mammalian orthoreovirus from least horseshoe bat in China. PLoS One. 2015. doi: 10.1371/journal.pone.0118598 25781475 PMC4364601

[18] Dandár E, Bálint Á, Kecskeméti S, Szentpáli-Gavallér K, Kisfali P, Melegh B, et al. Detection and characterization of a divergent avian reovirus strain from a broiler chicken with central nervous system disease. Arch Virol. 2013. doi: 10.1007/s00705-013-1739-y 23771766

[19] Rosa ACG, Ferreira HL, Gomes DE, Táparo CV, Cardoso TC. Isolation and molecular characterization of Brazilian turkey reovirus from immunosuppressed young poults. Arch Virol. 2014. doi: 10.1007/s00705-013-1947-5 24327096 PMC7086608

[20] Troxler S, Rigomier P, Bilic I, Liebhart D, Prokofieva I, Robineau B, et al. Identification of a new reovirus causing substantial losses in broiler production in France, despite routine vaccination of breeders. Veterinary Record. 2013. doi: 10.1136/vr.101262 23636701

[21] Mor SK, Verma H, Sharafeldin TA, Porter RE, Jindal N, Ziegler A, et al. Characterization of S class gene segments of a newly isolated turkey arthritis reovirus. Virology. 2014. doi: 10.1016/j.virol.2014.06.009 25043587

[22] Ide PR, Dewitt W. Serological incidence of avian reovirus infection in broiler-breeders and progeny in Nova Scotia. Canadian Veterinary Journal. 1979. 231481 PMC1789636

[23] de Gussem J, Swam H, Lievens K, de Herdt P. Reovirus tenosynovitis in a flock of layer breeders. Avian Pathology. 2010. doi: 10.1080/03079451003717597 20544421

[24] Howell SH, Walker LL. Synthesis of DNA in toluene-treated Chlamydomonas reinhardi (DNA replication-chloroplast DNA-cell cycle-electron microscopy). Proc Natl Acad Sci U S A. 1972. doi: 10.1073/pnas.69.2.490 4621835 PMC426487

[25] Bezerra DAM, da Silva RR, Kaiano JHL, Silvestre RVD, de Souza Oliveira D, Linhares AC, et al. Detection of avian group D rotavirus using the polymerase chain reaction for the VP6 gene. J Virol Methods. 2012. doi: 10.1016/j.jviromet.2012.07.017 22820073

[26] Palya V, Glávits R, Dobos-Kovács M, Ivanics É, Nagy E, Bányai K, et al. Reovirus identified as cause of disease in young geese. Avian Pathology. 2003. doi: 10.1080/030794502100007187 12745366

[27] Giangaspero M, Vanopdenbosch E, Nishikawa H, Tabbaa D. Prevalence of antibodies against respiratory viruses (parainfluenza virus type 3, respiratory syncytial virus, reovirus and adenovirus) in relation to productivity in Syrian awassi sheep. Trop Anim Health Prod. 1997. doi: 10.1007/BF02632323 9203308

[28] Taber R, Alexander V, Whitford W. Persistent reovirus infection of CHO cells resulting in virus resistance. J Virol. 1976. doi: 10.1128/JVI.17.2.513-524.1976 176428 PMC515443

[29] Baroni A, Bertoncin P, D’aprile PN, Felluga B. Ultrastructural histopathology of chick embryo chorioallantoic membrane infected with an avian reovirus. Avian Pathology. 1980. doi: 10.1080/03079458008418418 18770272

[30] Petek M, D’Aprile PN, Cancellotti F. Biological and physico-chemical properties of the infectious bursal disease virus (IBDV). Avian Pathology. 1973. doi: 10.1080/03079457308418073 18777392

[31] Rey MEC, D’Andrea E, Calvert-Evers J, Paximadis M, Boccardo G. Evidence for a phytoreovirus associated with tobacco exhibiting leaf curl symptoms in South Africa. Phytopathology. 1999. doi: 10.1094/PHYTO.1999.89.4.303 18944775

[32] Vindevogel H, Meulemans G, Pastoret PP, Schwers A, Calberg-Bacq CM. Reovirus infection in the pigeon. Annales de Recherches Veterinaires. 1982. 6301348

[33] Guy JS, Levy MG, Ley DH, Barnes HJ, Gerig TM. Experimental reproduction of enteritis in bobwhite quail (Colinus virginianus) with Cryptosporidium and reovirus. Avian Dis. 1987. doi: 10.2307/1591021 2831866

[34] Magee DL, Montgomery RD, Maslin WR, Wu Ching Ching, Jack SW. Reovirus associated with excessive mortality in young bobwhite quail. Avian Dis. 1993. doi: 10.2307/1591925 8141744

[35] Ritter GD, Ley DH, Levy M, Guy J, Barnes HJ. Intestinal cryptosporidiosis and reovirus isolation from bobwhite quail (Colinus virginianus) with enteritis. Avian Dis. 1986. doi: 10.2307/1590430 3021104

[36] Huhtamo E, Uzcátegui NY, Manni T, Munsterhjelm R, Brummer-Korvenkontio M, Vaheri A, et al. Novel orthoreovirus from diseased crow, Finland [10]. Emerging Infectious Diseases. 2007. doi: 10.3201/eid1312.070394 18258067 PMC2876751

[37] Lawson B, Dastjerdi A, Shah S, Everest D, Núñez A, Pocknell A, et al. Mortality associated with avian reovirus infection in a free-living magpie (Pica pica) in Great Britain. BMC Vet Res. 2015. doi: 10.1186/s12917-015-0329-5 25880683 PMC4336486

[38] Curtis PE, al-Mufarrej SI, Jones RC, Morris J, Sutton PM. Tenosynovitis in young pheasants associated with reovirus, staphylococci and environmental factors. Vet Rec. 1992. doi: 10.1136/vr.131.13.293 1332244

[39] Lu H, Tang Y, Dunn PA, Wallner-Pendleton EA, Lin L, Knoll EA. Isolation and molecular characterization of newly emerging avian reovirus variants and novel strains in Pennsylvania, USA, 2011–2014. Sci Rep. 2015. doi: 10.1038/srep14727 26469681 PMC4606735

[40] Lloréns-Rico V, Serrano L, Lluch-Senar M. Assessing the hodgepodge of non-mapped reads in bacterial transcriptomes: real or artifactual RNA chimeras? BMC Genomics. 2014;15: 633. doi: 10.1186/1471-2164-15-633 25070459 PMC4122791

[41] Kumar S, Stecher G, Peterson D, Tamura K. MEGA-CC: Computing core of molecular evolutionary genetics analysis program for automated and iterative data analysis. Bioinformatics. 2012. doi: 10.1093/bioinformatics/bts507 22923298 PMC3467750

[42] Goldenberg D, Pasmanik-Chor M, Pirak M, Kass N, Lublin A, Yeheskel A, et al. Genetic and antigenic characterization of sigma C protein from avian reovirus. Avian Pathology. 2010. doi: 10.1080/03079457.2010.480969 20544425

[43] Bányai K, Dandár E, Dorsey KM, Mató T, Palya V. The genomic constellation of a novel avian orthoreovirus strain associated with runting-stunting syndrome in broilers. Virus Genes. 2011. doi: 10.1007/s11262-010-0550-z 21116842

[44] Dandár E, Huhtamo E, Farkas SL, Oldal M, Jakab F, Vapalahti O, et al. Complete genome analysis identifies Tvärminne avian virus as a candidate new species within the genus Orthoreovirus. Journal of General Virology. 2014. doi: 10.1099/vir.0.060699-0 24421111

[45] Davis JF, Kulkarni A, Fletcher O. Reovirus infections in young broiler chickens. Avian Dis. 2013. doi: 10.1637/10515-021313-Case.1 24689195

[46] Mor SK, Sharafeldin TA, Porter RE, Goyal SM. Molecular characterization of L class genome segments of a newly isolated turkey arthritis reovirus. Infection, Genetics and Evolution. 2014. doi: 10.1016/j.meegid.2014.07.012 25057811

[47] Sharafeldin TA, Mor SK, Sobhy NM, Xing Z, Reed KM, Goyal SM, et al. A newly emergent Turkey arthritis reovirus shows dominant enteric tropism and induces significantly elevated innate antiviral and T helper-1 cytokine responses. PLoS One. 2015. doi: 10.1371/journal.pone.0144085 26659460 PMC4684236

[48] Payne S. Family Reoviridae. Viruses. 2017. doi: 10.1016/b978-0-12-803109-4.00026-x

[49] Borsa J, Sargent MD, Lievaart PA, Copps TP. Reovirus: Evidence for a second step in the intracellular uncoating and transcriptase activation process. Virology. 1981;111: 191–200. doi: 10.1016/0042-6822(81)90664-4 7233831

[50] Liu HJ, Lee LH, Hsu HW, Kuo LC, Liao MH. Molecular evolution of avian reovirus: Evidence for genetic diversity and reassortment of the S-class genome segments and multiple cocirculating lineages. Virology. 2003. doi: 10.1016/s0042-6822(03)00415-x 14517086

[51] McElroy K, Zagordi O, Bull R, Luciani F, Beerenwinkel N. Accurate single nucleotide variant detection in viral populations by combining probabilistic clustering with a statistical test of strand bias. BMC Genomics. 2013. doi: 10.1186/1471-2164-14-501 23879730 PMC3848937

[52] Kuroda M, Katano H, Nakajima N, Tobiume M, Ainai A, Sekizuka T, et al. Characterization of Quasispecies of Pandemic 2009 Influenza A Virus (A/H1N1/2009) by De Novo Sequencing Using a Next-Generation DNA Sequencer. PLoS One. 2010. doi: 10.1371/journal.pone.0010256 20428231 PMC2859049

[53] Kwok H, Tong AHY, Lin CH, Lok S, Farrell PJ, Kwong DLW, et al. Genomic sequencing and comparative analysis of Epstein-Barr virus genome isolated from primary nasopharyngeal carcinoma biopsy. PLoS One. 2012. doi: 10.1371/journal.pone.0036939 22590638 PMC3349645

