# Supplementary material for: Genetic characterization of a novel pheasant-origin orthoreovirus using Next-Generation Sequencing
Source: PLoS One. 2022 Nov 21;17(11):e0277411. doi: 10.1371/journal.pone.0277411 (PMC9678273; doi:10.1371/journal.pone.0277411)
Supplement: S1 Table — (DOCX) [file pone.0277411.s001.docx]

S1 Table. Genbank accession numbers of full genome segments of avian orthoreovirus (ARV) reference strains: PA05682, S1133, 138, 1733, 176, J18, BRoV, BRV, NBV, PuV, MRV1, MRV2 and MRV3

| **Segment** | **PA05682** | **S1133** | **138** | **1733** | **PA22342** | **J18** | **BRoV** | **BRV** | **NBV** | **PuV** | **MRV1** | **MRV2** | **MRV3** |
| --- | --- | --- | --- | --- | --- | --- | --- | --- | --- | --- | --- | --- | --- |
| **L1** | KM877325 | KF741756 | EU707933 | KF741706 | KP173683 | JX478260 | NC 014236 | NC 015877 | JF342674 | JF342666 | AF129820 | GU196308 | GQ468270 |
| **L2** | KM877326 | KF741757 | EU707935 | KF741707 | KP173684 | JX478261 | NC 015878 | NC 015878 | JF342673 | JF342667 | AF378003 | GU196307 | GQ468267 |
| **L3** | KM877327 | KF741758 | EU707937 | KF741708 | KP173685 | JX478262 | NC 014238 | HQ847905 | JF342672 | JF342668 | AF378003 | GU196306 | GQ468266 |
| **M1** | KM877328 | KF741759 | AY557188 | KF741709 | KP173686 | JX478263 | NC 014239 | HQ847906 | JF342676 | JF342669 | X59945 | GU196309 | GQ468268 |
| **M2** | KM877329 | KF741760 | AY557189 | KF741710 | KP173687 | JX478264 | NC 014240 | HQ847907 | JF342675 | JF342670 | AF490617 | GU196310 | GQ468269 |
| **M3** | KM877330 | KF741761 | AY557190 | KF741711 | KP173688 | JX478265 | NC 014241 | HQ847908 | JF342674 | JF342671 | AF174382 | GU196314 | GQ468271 |
| **S1** | KM877331 | KF741762 | AF218359 | KF741712 | KP173689 | JX478266 | NA* | NA | AF218360 | AY357730 | KF013857 | KF013857 | GQ468272 |
| **S2** | KM877332 | KF741763 | AF059717 | KF741713 | KP173690 | JX478267 | NC 014242 | AF059719 | AF059718 | AY357731 | REO1MCPS2A | GU196311 | GQ468273 |
| **S3** | KM877333 | KF741764 | AF059721 | KF741714 | KP173691 | JX478268 | NC 014243 | AF406787 | AF059722 | AY357732 | REOMCPS4 | GU196313 | GQ468275 |
| **S4** | KM877334 | KF741765 | AF059725 | KF741715 | KP173692 | JX478269 | NC 014244 | AF059727 | AF059726 | AY357733 | KF013858 | GU196312 | GQ468274 |

NA: sequence not available in Genbank
