# Supplementary material for: Genetic characterization of a novel pheasant-origin orthoreovirus using Next-Generation Sequencing
Source: PLoS One. 2022 Nov 21;17(11):e0277411. doi: 10.1371/journal.pone.0277411 (PMC9678273; doi:10.1371/journal.pone.0277411)
Supplement: Attachment — Submitted filename: Response letter to Editor and Reviewers_4-18-22.docx [file pone.0277411.s002.docx]

Date:	April 18, 2022

From:	Huaguang Lu, Corresponding author

To:	PLOS ONE Editors and Reviewers

Subj.:	Responses to Editor and Reviewers’ comments

Dear Editors and Reviewers,

A point-by-point response to each of the Editor and Reviewers’ comments/questions is as bellow in highlighted yellow:

I. **A** **Point-by-Point Response to Editor**

Journal Requirements:

When submitting your revision, we need you to address these additional requirements.

1. Please ensure that your manuscript meets PLOS ONE's style requirements, including those for file naming. The PLOS ONE style templates can be found at

https://journals.plos.org/plosone/s/file?id=wjVg/PLOSOne_formatting_sample_main_body.pdf and [https://journals.plos.org/plosone/s/file?id=ba62/PLOSOne_formatting_sample_title_authors_affiliations.pdf](https://nam10.safelinks.protection.outlook.com/?url=https%3A%2F%2Fjournals.plos.org%2Fplosone%2Fs%2Ffile%3Fid%3Dba62%2FPLOSOne_formatting_sample_title_authors_affiliations.pdf&data=04%7C01%7Chxl15%40psu.edu%7C0b04524adfc24d4d72c208d9f09cd1e8%7C7cf48d453ddb4389a9c1c115526eb52e%7C0%7C0%7C637805379789304618%7CUnknown%7CTWFpbGZsb3d8eyJWIjoiMC4wLjAwMDAiLCJQIjoiV2luMzIiLCJBTiI6Ik1haWwiLCJXVCI6Mn0%3D%7C3000&sdata=HIltwHpHONYlRrok3QElgzMqN4q61GrWZZf4%2BMpgA3Q%3D&reserved=0).

- Yes, we have revised this manuscript according to PLOS ONE's style requirements.

2. In your Methods section, please provide additional details regarding the animal material used in your study and ensure you have described the source. For more information regarding PLOS' policy on materials sharing and reporting, see <https://journals.plos.org/plosone/s/materials-and-software-sharing#loc-sharing-materials> ."

- Yes, additional information was added, lines 89 - 96.

3. Thank you for stating the following in the Acknowledgments Section of your manuscript:

“This research study was supported by Tianjin Ringpu Bio-Technology Co, Ltd., Tianjin, China.”

We note that you have provided additional information within the Acknowledgements Section that is not currently declared in your Funding Statement. Please note that funding information should not appear in the Acknowledgments section or other areas of your manuscript. We will only publish funding information present in the Funding Statement section of the online submission form.

Please remove any funding-related text from the manuscript and let us know how you would like to update your Funding Statement. Currently, your Funding Statement reads as follows:

“The authors have declared that no competing interests exist.”

Please include your amended statements within your cover letter; we will change the online submission form on your behalf.

- No Acknowledgments.
- No competing interests exist.

4. Please include your tables as part of your main manuscript and remove the individual files. Please note that supplementary tables (should remain/ be uploaded) as separate "supporting information" files.

- Yes, this revised manuscript includes all the 3 tables in one MS file.

Additional Editor Comments:

Major revision is required. Authors should address to all comments and concerns mentioned by reviewers and revise the paper in response to their advice. If the revision is completed successfully, I will reconsider my decision.

- Yes, we have done the major revision accordingly,
- This revised manuscript has been edited by professional editors at Editage ([www.editage.com](http://www.editage.com)) to ensure language and grammars accuracy,

[Note: HTML markup is below. Please do not edit.]

Reviewers' comments:

Reviewer's Responses to Questions

 5. Review Comments to the Author

Please use the space provided to explain your answers to the questions above. You may also include additional comments for the author, including concerns about dual publication, research ethics, or publication ethics. (Please upload your review as an attachment if it exceeds 20,000 characters) - no additional comments.

**II.	A Point-by-Point Response to Reviewers**

Reviewer #1: The Authors investigated an interesting topic concerning the ARV infections. Authors described field strain (Reo/SDWF/Pheasant/17608/20) of avian orthoreovirus (ARV), which have been isolated from a flock of ringneck pheasants in Weifang district of Shandong Province, China. This strain was genetically characterized being a novel field variant strain in conducting whole genome sequencing by using Next-Generation Sequencing (NGS) technique on Illumina MiSeq platform.
The de novo assembly of the ARV reads resulted in generation of 10 ARV-related contigs with the average sequencing coverage from 1390× to 1977× according to 10 ARV genome segments. The complete genomes of this pheasant-origin ARV (Reo/SDWF /Pheasant/17608/20) were 23,495 bp in length and consist of 10 dsRNA segments ranged from 1192 bp (S4) to 3958 bp (L1) encoding 12 viral proteins.
The manuscript was properly structured. The Introduction section was clearly written. The Material and methods section described the ARV isolation from tendon tissue of a pheasant case with hepatopathy symptoms and RT-PCR amplification and Sanger sequencing have been performed. The next step of the studies Next-generation sequencing have been performed. The results section well reported.
However, some changes are necessary before a possible acceptance of the paper.
Here, I am reporting some suggestions aiming to further improve the paper:

Comments:
1. The English grammar needs proofread

- This revised manuscript has been edited by professional editors at Editage ([www.editage.com](http://www.editage.com)) to ensure language and grammars accuracy,

2. Line 336, please use the reference

As reoviral infections usually appear in the poultry flocks with an lover immune status, where there is also infection with other viruses that reduce immunity, please mention it and add the references below.
Fowl adenovirus strains 1/A and 11/D isolated from birds with reovirus infection.
Niczyporuk JS, Kozdrun W, Czekaj H, Stys-Fijol N. PLoS One. 2021 Aug 19;16(8):e0256137. doi: 10.1371/journal.pone.0256137. eCollection 2021. PMID: 34411166 Free PMC article.

- Thank you, we revised this paragraph in using the reference.
- The conserved regions at the 5’ and 3’ ends of the positive strand are the same as the ARV members, but there are some differences at the 5' end with the non-ARV reference strains, indicating that the genome segments of SDWF17608 strain were all classified into ARV species and did not undergo fragment reassortment with non-ARV strains (Mertens et al., 2000).

3. Concerning the Fig. 3 - you have to improve the figure, because is almost impossible to read it.
The data I found interesting and the results connected with full genome sequencing for this novel pheasant ARV were interesting from even the epidemiological point of view and the use of bioinformatic tools to perform NGS analyzes has become useful for determining the evolutionary relationships between the studied ARV strain!

- Yes agree, Fig 3 has been revised.

Reviewer #2: The authors report the isolation and genetic characterization of an avian orthoreovirus strain from a pheasant.

Overall, reports about “new” viruses bear always a certain scientific value and are worth to be published. However, the actual manuscript is very poorly written with many grammatical mistakes. The English of the manuscript needs to be improved by someone with high proficiency to target the grammatical errors and to increase the overall language clarity.

- This revised manuscript has been edited by professional editors at Editage ([www.editage.com](http://www.editage.com)) to ensure language and grammars accuracy,

Introduction:
The introduction is way too long. Therefore, it shall be shortened and more focused on the scientific objectives of the manuscript.

- Yes, some revisions were done on the introduction.

Variant strain is a redundant term. A virus strain is a variant that possesses unique and stable phenotypic characteristics.
Pheasants, which are the targeted bird species of the manuscript, are not classified as wild birds, but as game birds.

- Yes agree, corrections were made,

Material & Methods:
After building up the readers interest in the introduction highlighting clinical features of the Reovirus infection and the importance of birds other than commercial poultry as reservoirs for ARVs, the manuscript fails to provide information clinical data regarding the infected pheasant: origin, husbandry system, flock size, mortality, morbidity, clinical signs and also post-mortem and histological findings. Why was tendon tissue used for isolation? Did the birds suffered from tenosynovitis?
The term “symptom” is only used in Human Medicine. In Veterinary Medicine the term Clinical Signs is used instead.
Did the clinical signs lasted for 2 weeks?
Was the RNA extracted from the original sample or from ARV propagated in cell culture?
The manuscript should be more clear about the samples used for RNA extraction. The only explanation I can see for the presence of chicken RNA in a pheasant-origin sample is the use of the cell culture propagated ARV for RNA extraction.

- Yes agree, additional information has been added to address these questions (Line 89-108), thank you for these great comments.

Discussion:
The discussion section of this manuscript is very poor. The language is not clear, concise and needs to be corrected grammatically by a proficient speaker.
It is expected that the key-findings of the experiment are summarized and explained in a systematic manner in the context of previous studies leading to deductions and/or hypotheses. The manuscript fails at that, using only selected data for discussion but in confused manner with confusing language.
There is no mention of demarcation criteria for ARV genomes, which is surprising after extensive sequence identity and phylogenetic analysis.
Some references (line 333) might be substituted with a decent review or book chapter.
Lines 352-354: This sentence is not correct. The homology between σB genes is much higher than among σC genes.
References:
The reference list is too long with 57 references. Many of the references should be substituted by a recent review paper or the chapter in Diseases of Poultry, which it was not listed as reference. Additionally, the ICTV chapter on Reoviridae was recently updated and should also be used as reference.

- The discussion section has been revised; accordingly, much appreciate these excellent comments for improvement of this manuscript.

Reviewer #3: PONE-D-21-27563
Title: Genetic characterization of a novel pheasant origin orthoreovirus using next generatio sequencing
Corresponding author: Huaguang Lu

This manuscript summarizes the genetic characterization of a novel Pheasant ARV. While the work is well done and described, there are some concerns about sequencing of segmented viruses and the possibility of mixed isolates. Starting with a pure isolate is a must when you want to characterize a virus (especially when this virus is segmented). In addition, if no info is available on the potential mixed nature of the virus is very difficult to know if the sequences obtained from each of the genes match with the other genes obtained. It wasn’t clear if diverse sequences were obtained for each gene. This situation at least needs to be clarified, explained and discussed in this paper. More information about the origin of the virus is needed and its impacts on the flocks where it was retrieved. The last information will help associate findings with potential use of the information in preventing issues caused by this specific virus. Finally, there are passages of the manuscript where grammar needs to be revised for clarity. Verbal tenses and sentence construction could be improved. Discussion overall could be improved.

- Yes agree, additional information for ARV isolation has been added to address these questions (Line 89 - 108),

ABSTRACT
It is common in reovirus isolates to obtain more than one reovirus or several different viruses including adeno, rota, parvo on the isolates. Was that something that was encountered in your work? If several Reo strains were detected how you know which segments match to form the strain you are molecularly characterizing?

- Yes certainly, the other common avian viruses were tested negative, a brief information of the ARV isolate was added in Material and Methods section, the first paragraph. ARV positive isolations were made in both tendon pool and intestine pool specimens, but only the ARV isolate from tendon pool was used in this study, because which should be relatively pure ARV isolate.

INTRODUCTION
Line 50. Please revise sentence for clarity,
Line 54. Check grammar
Some passages require grammar revision

- This revised manuscript has been edited by professional editors at Editage ([www.editage.com](http://www.editage.com)) to ensure language and grammars accuracy,

MATERIALS AND METHODS
Lines 99 to 105. Was the virus isolate plaque purified? This would be important if you want to characterize a specific virus. As mentioned above there is a high percent of isolates that possess multiple reoviruses and even other viruses.

- Yes agree, it’s the best to get done virus purification, however, due to our limited lab condition, we choose to use the ARV isolate from tendon tissues in this study, which should be relatively free from other avian viruses.

Line 129. Should say “was loaded”, - yes corrected (Line 130 on this revised manuscript)
NGS doesn’t allow to match the right pieces into an existent Reo gene if there are more than one reovirus present in the sample. That is why is crucial to know if the original isolate was pure (only on reovirus was detected)
Which ARV was used as reference for the construction of the full genome?

- de novo assembly assay was used to construct the virus genome and the genome was upgraded by mapping back all reads to the de novo assembly genome.

Line 161 to 162. Not sure about this, what is the meaning that this genome was set as a scale?
What is the reference helping with?
Line 167 to 171. Was this sequencing done in China or the US and, are there any animal experiments involved? Why there is an IACUC approval?

- all the studies were done in China, the “Ethics Statement” was removed, no animal experiment was included in this study.

RESULTS
Lines 175 to 181. Please review sentences and check grammar. In its present form is difficult to understand. In addition, be clear with the results that are being shown. In addition to GenBank numbers refer to relevant data to compare homologies
Images are extremely low quality, they are very difficult to read, please correct
Were all sequences retrieved from each gene similar? If so, how similar they were in terms of homologies?
Figure 4 is not in the manuscript. I will need in order to review this section of the manuscript.

- These corrections have been made accordingly

DISCUSSION
Line 336 should say “species” says “spices” – corrected, thank you for the detail review!
Discussion is somewhat unorganized and do not cover the issue of full genome sequencing segmented viruses. – Yes we have revised the discussion.

Reviewer #4: Tang et al. grew a virus from an infected pheasant in a cell line of avian origin, sequence the resulting S1 gene by Sanger sequencing and then the whole genome by Illumina. The obtained sequence is compared to available sequences to conclude that this virus has some gene segment rearrangements.
General comment: The study is of little interest as the sequence is from just one animal and the virus was not directly sequenced, but first grown in a cell line, so the virus sequenced might differ from the original one. The results obtained are of little, if any, interest. Also, it is beyond my understanding why they grow the virus, make DNA copies of the genome segment S1 and perform Sanger sequencing and afterwards go to Illumina for the rest 9 segments while Sanger is more accurate: what is the reason?

Thank you for your general comment.

- We have realized that the manuscript did not contain sufficient information of the pheasant cases, which probably cause some misunderstanding. We have added the case information in the revised manuscript.
- The ARV isolation was made tend pool from 10 sick peasant chicks,
- Virus isolation is always the best to certain the disease agent, especially a viral pathogen.
- Virus isolation can be the propagation and purification process for the causative virus pathogen, not to change the virus.
- Although it’s true that molecular tests can be applied directly on clinical specimens, an isolated virus is always preferred for further studies, including the molecular sequencing assays.

Particular observations:
- In the “Introduction” section, the taxonomical data is out of date: the references given are 20 years old at least and in fact there are more than 5 species in the Orthoreovirus genus.
- If they want to be accurate in protein listing, there are some missing proteins that are formed inside the infected cell by post-translational processing (muBC, muBN...).
- No description at all is given of the method used for virus isolation. Also, the antibody used is not described, it is instead said that it was characterized on a previous study, but the same reference is given on such study: it should at least be said if the antibody was raised against the whole, virus, a particular protein, group of proteins, etc.
- Fig 1 is prescindible: just data from their methodology that is not either informative or useful at all.
- Fig 2 Why this comparison of just p10 and p17? Why not then sigmaC that is supposed to be the most variable? I don´t see any reason for this figure neither.
- The references are just a nonsense: there are many missing articles. Just to cite some, those describing the tricistronic nature of the S1 gene (discussion of that deserves figure 2 to the authors). On the other hand, several others are no cited on the text, 35 for example, something that makes sense as it has nothing to do with the subject of this paper.

- Thank you for these comments, however, we hardly agree with, 1) since we have written this reteach manuscript but not a review article, so we have listed the references we only used, a good reference can be forever no matter how old; 2) This study is focused on genetic characterization studies, we think it should be better to write briefly on virus isolation serologic tests; 3) Fig 1 (Results of the homology searches among next-generation sequencing) provides the results of (NGS) reads and sequencing coverage analysis, Fig 2 (Amino acid alignment of the p10 and p17 proteins) provides the particular comparison results of this study ARV with other ARV strains.

Finally, we much appreciate every reviewer’s effort in providing these great comments and suggestions to make improvement of this manuscript.
