# Supplementary material for: Genetic characterization of a novel pheasant-origin orthoreovirus using Next-Generation Sequencing
Source: PLoS One. 2022 Nov 21;17(11):e0277411. doi: 10.1371/journal.pone.0277411 (PMC9678273; doi:10.1371/journal.pone.0277411)
Supplement: Attachment — Submitted filename: A Letter of Responses to Reviewers.docx [file pone.0277411.s003.docx]

Date:	September 16, 2022

From:	Huaguang Lu, correspondence author

To:	Editor and Reviewers

Subj.:	Responses to Reviewers

Dear Editor and Reviewers,

Thank you for your comments and suggestions to revise this manuscript to be acceptable for publication. We have revised the manuscript accordingly, and below is our point-by-point response (blue-highlighted) to each of the points made by the Editor and / or Reviewers.

**Journal Requirements:**

Please review your reference list to ensure that it is complete and correct. If you have cited papers that have been retracted, please include the rationale for doing so in the manuscript text, or remove these references and replace them with relevant current references. Any changes to the reference list should be mentioned in the rebuttal letter that accompanies your revised manuscript. If you need to cite a retracted article, indicate the article’s retracted status in the References list and also include a citation and full reference for the retraction notice.

**Authors’ response:** yes agree, we have removed all previous references in the manuscript text and the reference list, we have replaced with reformatted references in the manuscript text and the reference list in accordance with the PLOS One Journal Requirements.

**Additional Editor Comments (if provided):**

Authors need to address following concern from a reviewer-----
"The author used a virus isolated from tendons to perform sequencing and genetically characterize this virus. If plaque purification was not done a chance of mixing sequences from different viruses is a possibility. I still don't see comments and discussion related to this. Please highlight and properly point lines where this topic is addressed"

**Authors’ response:** Please accept our apology for missing this comment in the R1 version.

Yes agree, it would be good if we could get done the plaque purification on this AIV isolate to certain not containing other viruses. Alternatively, we conducted subsequent tests on this ARV isolate and confirmed that it is negative for other common avian viruses including AIV, NDV, FAV and rotavirus. Also, in the NGS analysis, except for the contigs of ARV, draft contigs were not mapped to other-related viruses. Thus, we considered this pheasant ARV isolate should be rare chance in mixing with other viruses.

In the “**Materials and Methods**” section of this revised version(R2), we have added additional information of this pheasant ARV isolate in the highlighted yellow as the following,

Lines 104-105: The ARV isolation was made in LMH cell cultures and produced giant or bloom-like cytopathic effect (CPE) cells, which were characteristic to ARV and confirmed positive for ARV by fluorescent antibody (FA) test

Lines 104-105: The ARV cell culture material was subsequently tested negative for other avian viruses which could cause the pheasant leg lesions such as avian influenza virus, Newcastle disease virus, fowl adenovirus type 1 and rotavirus.

In the “**Results**” section of this revised version(R2), we added additional Sanger sequencing result as,

Lines 172-174: In the next-generation sequencing analysis, the contigs of ARV were clearly mapped, whereas draft contigs to other-related viruses were not mapped.

[Note: HTML markup is below. Please do not edit.]

Reviewers' comments:

Reviewer's Responses to Questions

**Comments to the Author**

1. If the authors have adequately addressed your comments raised in a previous round of review and you feel that this manuscript is now acceptable for publication, you may indicate that here to bypass the “Comments to the Author” section, enter your conflict of interest statement in the “Confidential to Editor” section, and submit your "Accept" recommendation.

Reviewer #1: All comments have been addressed

Reviewer #3: (No Response)

Reviewer #5: All comments have been addressed

2. Is the manuscript technically sound, and do the data support the conclusions?

The manuscript must describe a technically sound piece of scientific research with data that supports the conclusions. Experiments must have been conducted rigorously, with appropriate controls, replication, and sample sizes. The conclusions must be drawn appropriately based on the data presented.

Reviewer #1: Yes

Reviewer #3: Yes

Reviewer #5: Yes

3. Has the statistical analysis been performed appropriately and rigorously?

Reviewer #1: N/A

Reviewer #3: Yes

Reviewer #5: Yes

4. Have the authors made all data underlying the findings in their manuscript fully available?

The [PLOS Data policy](https://nam10.safelinks.protection.outlook.com/?url=http%3A%2F%2Fwww.plosone.org%2Fstatic%2Fpolicies.action%23sharing&data=05%7C01%7Chxl15%40psu.edu%7C6ef52aaa3bfd43fe1c3308da73e384a3%7C7cf48d453ddb4389a9c1c115526eb52e%7C0%7C0%7C637949718272198072%7CUnknown%7CTWFpbGZsb3d8eyJWIjoiMC4wLjAwMDAiLCJQIjoiV2luMzIiLCJBTiI6Ik1haWwiLCJXVCI6Mn0%3D%7C3000%7C%7C%7C&sdata=Q%2B5H%2BIGW4YzVxX2s%2F%2Bp8HF72rMwGGLJuHTIKDthV030%3D&reserved=0) requires authors to make all data underlying the findings described in their manuscript fully available without restriction, with rare exception (please refer to the Data Availability Statement in the manuscript PDF file). The data should be provided as part of the manuscript or its supporting information, or deposited to a public repository. For example, in addition to summary statistics, the data points behind means, medians and variance measures should be available. If there are restrictions on publicly sharing data—e.g. participant privacy or use of data from a third party—those must be specified.

Reviewer #1: Yes

Reviewer #3: Yes

Reviewer #5: Yes

5. Is the manuscript presented in an intelligible fashion and written in standard English?

PLOS ONE does not copyedit accepted manuscripts, so the language in submitted articles must be clear, correct, and unambiguous. Any typographical or grammatical errors should be corrected at revision, so please note any specific errors here.

Reviewer #1: Yes

Reviewer #3: Yes

Reviewer #5: Yes

6. Review Comments to the Author

Please use the space provided to explain your answers to the questions above. You may also include additional comments for the author, including concerns about dual publication, research ethics, or publication ethics. (Please upload your review as an attachment if it exceeds 20,000 characters)

Reviewer #1: Authors described field avian orthoreovirus strain (Reo/SDWF/Pheasant/17608/20) isolated from a flock of game pheasants in Weifang, Shandong Province. The next-generation sequencing (NGS) technique on the Illumina MiSeq platform to determine the sequence of reovirus strain have been used. Among 870,197 35–151-mer sequencing reads, 297,711 (34.21%) were ARV sequences. The complete genome of SDWF17608 of pheasant origin was 23,495 bp in length and comprised 10 dsRNA segments ranging from 1192 bp (S4) to 3958 bp (L1) encoding 12 viral proteins. The sequence comparison between the current strain and classic ARV reference strains revealed 58.1–100% nucleotide (nt) identities and 51.4–100% amino acid (aa) identities in genome coding genes. The 10 RNA segments had conserved termini at the 5’ (5’- 33 GCUUUU) and 3’ (UCAUC-3’) ends, which were identical to those of published ARV strains. Phylogenetic analysis revealed that the SDWF17608 pheasant ARV field strain was closely related to chicken ARV strains in seven genome segment genes but showed significant sequence divergence in the M1, M3, and S2 segments. These findings suggested that this field variant of pheasant origin was a divergent ARV strain and likely originated from the reassortment of different chicken ARV strains.
The above work presents a detailed molecular (nucleotide and amino acid) characterization of reovirus strains isolated from pheasants using NGS sequencing. The prepared figures and tables presented in the above work are clear, understandable and concise and essential and significantly enrich the content of the discussed work.
These studies and the results obtained will allow for better understanding / illustrating the evolution of the genome of ARV strains and phylogenetic connections that may occur in strains of reoviruses isolated from different species of birds. The work has a high cognitive value and applies for its publication in the PLOS one journal.

Reviewer #3: The author used a virus isolated from tendons to perform sequencing and genetically characterize this virus. If plaque purification was not done a chance of mixing sequences from different viruses is a possibility. I still don't see comments and discussion related to this. Please highlight and properly point lines where this topic is addressed.

**Authors’ response:** Please accept our apology for missing this comment in the R1 version.

Yes agree, it would be good if we could get done the plaque purification on this AIV isolate to certain not containing other viruses. Alternatively, we conducted subsequent tests on this ARV isolate and confirmed that it is negative for other common avian viruses including AIV, NDV, FAV and rotavirus. Also, in the NGS analysis, except for the contigs of ARV, draft contigs were not mapped to other-related viruses. Thus, we considered this pheasant ARV isolate should be rare chance in mixing with other viruses.

In the “**Materials and Methods**” section of this revised version(R2), we have added additional information of this pheasant ARV isolate in the highlighted yellow as the following,

Lines 104-105: The ARV isolation was made in LMH cell cultures and produced giant or bloom-like cytopathic effect (CPE) cells, which were characteristic to ARV and confirmed positive for ARV by fluorescent antibody (FA) test

Lines 104-105: The ARV cell culture material was subsequently tested negative for other avian viruses which could cause the pheasant leg lesions such as avian influenza virus, Newcastle disease virus, fowl adenovirus type 1 and rotavirus.

In the “**Results**” section of this revised version(R2), we added additional Sanger sequencing result as,

Lines 172-174: In the next-generation sequencing analysis, the contigs of ARV were clearly mapped, whereas draft contigs to other-related viruses were not mapped.

Reviewer #5: (No Response)

7. PLOS authors have the option to publish the peer review history of their article ([what does this mean?](https://nam10.safelinks.protection.outlook.com/?url=https%3A%2F%2Fjournals.plos.org%2Fplosone%2Fs%2Feditorial-and-peer-review-process%23loc-peer-review-history&data=05%7C01%7Chxl15%40psu.edu%7C6ef52aaa3bfd43fe1c3308da73e384a3%7C7cf48d453ddb4389a9c1c115526eb52e%7C0%7C0%7C637949718272198072%7CUnknown%7CTWFpbGZsb3d8eyJWIjoiMC4wLjAwMDAiLCJQIjoiV2luMzIiLCJBTiI6Ik1haWwiLCJXVCI6Mn0%3D%7C3000%7C%7C%7C&sdata=Jc66tjJ1FErctDdulDuQ3hG1cbJlhDbV2Gz4e4HbeGo%3D&reserved=0)). If published, this will include your full peer review and any attached files.


If you choose “no”, your identity will remain anonymous but your review may still be made public.


**Do you want your identity to be public for this peer review?** For information about this choice, including consent withdrawal, please see our [Privacy Policy](https://nam10.safelinks.protection.outlook.com/?url=https%3A%2F%2Fwww.plos.org%2Fprivacy-policy&data=05%7C01%7Chxl15%40psu.edu%7C6ef52aaa3bfd43fe1c3308da73e384a3%7C7cf48d453ddb4389a9c1c115526eb52e%7C0%7C0%7C637949718272198072%7CUnknown%7CTWFpbGZsb3d8eyJWIjoiMC4wLjAwMDAiLCJQIjoiV2luMzIiLCJBTiI6Ik1haWwiLCJXVCI6Mn0%3D%7C3000%7C%7C%7C&sdata=Q263hMRnrdWt9Wt3L5Vyx0qsqCO7ZXCyoMGcNE8bAU0%3D&reserved=0).

Reviewer #1: **Yes:** Jowita Samanta Niczyporuk

Reviewer #3: No

Reviewer #5: No
